# Radiopharmaceuticals for Relapsed or Refractory Ovarian Cancers

**DOI:** 10.3389/fonc.2019.00180

**Published:** 2019-03-26

**Authors:** Charles A. Kunos, Jacek Capala, Shanda Finnigan, Gary L. Smith, Susan Percy Ivy

**Affiliations:** ^1^Cancer Therapy Evaluation Program, National Cancer Institute, Bethesda, MD, United States; ^2^Radiation Research Program, National Cancer Institute, Bethesda, MD, United States

**Keywords:** radiopharmaceutical, radiation, radionuclides, nuclear medicine, ovarian cancer

## Abstract

Targeted radiopharmaceuticals for therapeutic use deliver radionuclides directly to tumor anywhere in the body, and therefore, have renewed interest for clinical development in women with disseminated chemorefractory ovarian cancers. About two in every five women with advanced stage ovarian cancer outlive their disease after the first treatment phase, with the rest rendered incurable due to the chemorefractory nature of their disease. The National Cancer Institute (NCI) Cancer Therapy Evaluation Program conducted 67 phase I or phase Ib trials among women with relapsed or refractory ovarian cancer between 1989 and 2017 in an effort to uncover tolerable and effective drug combinations intended to increase survival rates. None of these early clinical development phase trials involved radiopharmaceuticals. Here, the NCI provides its perspective on targeted radiopharmaceutical conjugates alone or in combination with its experimental therapeutics portfolio for women with relapsed or refractory ovarian cancer. An infrastructure build for Federal radiopharmaceutical medical monitoring and adverse event reporting has begun.

## Introduction

In 2018, ovarian cancers collectively represent the tenth (2.5%) most common any-type cancer in American women (1). However, ovarian cancers are the fifth (5%) leading cause of cancer-related death in American women (1). This is mostly a consequence of the finding that four out of every five women with ovarian cancer present with advanced stage disease disseminating throughout the abdominal cavity at initial diagnosis (1). Half of all American women diagnosed with ovarian cancer are younger than age 63 years (1). First and subsequent phases of treatment for women with this disease undergo surgery followed by platinum-based chemotherapy, integrating second-look debulking surgeries for minimally chemotherapy responding disease under appropriate conditions. Five-year survival rates for such treated women might be as high as 73 percent when disease is confined to the abdominal cavity, but might be as low as 29 percent when disease metastasizes outside the abdominal cavity (1). For women with advanced stage ovarian cancer, the US National Cancer Institute (NCI) Cancer Therapy Evaluation Program has studied experimental therapeutic agents in 67 phase I or phase Ib trials between 1989 and 2017. All clinical development plans intended to raise overall disease remission and mortality rates. Through novel agent discovery, about one-half of women currently outlive their ovarian cancer long-term (2). Those women unable to achieve a disease-free status after initial phases of treatment, often due to occult chemorefractory disease in the abdomen, ultimately die of their disease (2).

A standard approach to the first phase of ovarian cancer treatment is abdominal surgery followed by carboplatin-paclitaxel chemotherapy (3). A novel approach for next-generation clinical trials seek to incorporate targeted radiopharmaceuticals that integrated into first-line treatment either to augment chemotherapy effects or to eradicate chemotherapy insensitive but radiation sensitive disease (4). There is historical precedence for such an approach. For example, intrabdominal instillation of the β-particle emitter ^32^P chromic phosphate was studied in four randomized clinical trials of women with advanced stage ovarian cancer (5–8). ^32^P chromic phosphate as a single infusion was well-tolerated and deemed clinically beneficial (5–8). The further clinical development of early radiopharmaceuticals was stopped due to clunky logistics for ^32^P chromic phosphate instillation and a clinical desire to test newer chemotherapy agents. Targeted α-particle radiopharmaceutical conjugates combine the affinity and specificity of molecular targeting with cytotoxic energy-rich radiation delivered anywhere in the body to cancer cells and their microenvironment for disease control (9). As expected, the radioactive payload of targeted α-particle radiopharmaceutical conjugates drives the efficacy and toxicity of these agents. But, there is also the possibility that the targeting ligand and cleaved/non-cleaved chemical linkers may also contribute to organ-specific toxicity. As NCI leads clinical development of radiopharmaceuticals in the US, it becomes increasingly important to collect and to report radiopharmaceutical-related adverse events in early phase clinical trials for treatment of patients with ovarian cancer or other targeted diseases.

Adverse event reporting, or colloquially toxicity reporting, is compulsory in human subject research to ensure research subject safety and to appreciate the safety profile of treatment agents alone or in combination (10). Existing methods like the NCI's Common Terminology Criteria for Adverse Events (CTCAE, version 5) are reliable and accurate for describing toxicities on a five-point scale based on clinical criteria. In trial reports, toxicity data are described often via a summary table of the number and the proportion of high-grade 3 (severe or medically significant), 4 (life-threatening), or 5 (lethal) toxicities during the observational timespan of a trial. But, these same tables include little to no data on the cumulative severity or onset of toxicities during the first or subsequent course of treatment. These factors might also factor into the determination of whether a treatment is deemed tolerable (11). Moreover, it is worthwhile to describe toxicities in relation to drug schedule or intensity (Table 1). In modern drug clinical development, acute toxicities are considered to arise over a brief timeframe after drug exposure and might be transient, reversible, or persistent (11). At the other extreme, chronic toxicities arise over a long time period and are considered persistent and unremitting, or intermittent and recurring. Chronic toxicities manifest late such as after the first phase or cycle of treatment (11). Cumulative toxicities intensify and arise after repeated phases of treatment exposure (11). Late toxicities result in subclinical manifestations that do not fit immediate, intermittent, or short-term adverse clinical events, but rather are evident over time such as after multiple phases of treatment (11). Radiopharmaceutical treatments introduce the need for a new term among these definitions—subacute toxicities. Subacute toxicities are adverse events that arise over an intermediate time period, such as 1–3 months post-therapy, and might be transient, reversible, or persistent in duration. An example could be radiation-induced pneumonitis manifesting as non-productive cough 3 months after initial radiation dose exposure.

**Table 1:** Toxicity definitions relative to radiopharmaceutical exposure.

**Effects**	**Time relative to radiopharmaceutical exposure**	**Time duration**	**Example**
Acute toxicity	Arises over a brief timeframe after radiopharmaceutical exposure	Transient, reversible, or persistent	Nausea
Subacute toxicity	Arises over an intermediate time period (e.g., 1 to 3 months posttherapy)	Transient, reversible, or persistent	Pneumonitis
Chronic toxicity	Arises over a long timeframe after radiopharmaceutical exposure	Persistent/unremitting, intermittent/recurring	Fibrosis
Cumulative toxicity	Arises and intensifies after repeated radiopharmaceutical exposure	Persistent/unremitting, intermittent/recurring	Watering eyes
Late toxicity	Arises over a long timeframe after repeated radiopharmaceutical exposure	Persistent/unremitting, intermittent/recurring	Marrow hypoplasia

For this article, the NCI provides its perspective on workflow for collecting and for reporting information about subacute toxicities and other categories of adverse events for the clinical development of radiopharmaceuticals. NCI's prior and possible future radiopharmaceutical experience in the treatment of patients with advanced stage relapsed or refractory ovarian cancer provides context for this workflow.

## Challenges And Opportunities

Adapting NCI's CTCAE criteria either for the presence/absence of toxicity or for its grading of severity presents challenges to radiopharmaceutical clinical development. The NCI recognizes five discrete categories for any given CTCAE term that radiopharmaceutical-attributed toxicity must fit (Table 2) (10)—(A) laboratory/biomarker based toxicity that requires equipment to detect (like anemia, leukopenia, neutropenia, or thrombocytopenia); (B) observable/measurable toxicity that requires technical training to delineate (like eye examination for tearing caused by corneal or limbic irritation); (C) primarily subjective toxicity without observable components (like radiation-induced nausea); (D) primarily subjective toxicity with observable components (like radiation-induced diarrhea); and (E) primarily observable toxicity with subjective components (like radiation-induced alopecia). NCI considers categories A and B, as it applies to radiopharmaceuticals, to follow the established generic CTCAE terminology and grading of severity. This is because category A and B toxicities require either radiotherapy-independent clinical expertise for evaluation, or, technical equipment. Category E toxicities lend themselves to be identified by patients but require clinical expertise to assign severity and follow the established generic CTCAE terminology and grading of severity. Categories C and D toxicities have elements of frequency, severity, or interference with usual or daily activities noticed by patients (10). Thus, these categories of toxicity might be amenable to study by electronic patient-reported outcome measures, in a pilot or a formal trial project, rather than just description by generic CTCAE criteria (10). For instance, pretherapy severity of constipation prior to radium-223 administration might be important to capture using a patient-reported outcome-CTCAE (PRO-CTCAE) method. This is because radium-223 is actively eliminated from the body via the large intestine. Any delay in stool evacuation might intensify the frequency, severity, or interference of bowel toxicity as protracted bowel dwell time of radium-223 irradiates a longer length of radiation-sensitive bowel. For radiopharmaceuticals, category C or D toxicity can be anticipated due to prior clinical observations with conventional radiotherapy. Thus, NCI might plan to collect select category C or D toxicity (like constipation) as an adverse event of special interest. An adverse event of special interest identifies a toxicity for which an expedited adverse event report must be filed to the NCI in its sponsored trials (12). The NCI remains willing to lead clinical development of targeted radiopharmaceutical conjugates because elements of its clinical trial enterprise, such as medical monitoring and safety data reporting, are efficient and cost-effective for such endeavors now and in the foreseeable future. However, unique challenges arise, and these challenges can be appreciated in the context of radiopharmaceutical clinical use in women with advanced stage ovarian cancer.

**Table 2:** Common terminology criteria for adverse event (CTCAE) categories for radiopharmaceuticals.

**Category**	**Item**	**Example**
Laboratory/Biomarker	Requires equipment	Lymphocyte count decreased (A finding based on laboratory test results that indicate a decrease in number of lymphocytes in a blood specimen.)
Observable/Measurable	Requires technical skill	Watering eyes (A disorder characterized by excessive tearing in the eyes; it can be caused by overproduction of tears or impaired drainage of the tear duct.)
Primarily subjective without observable component	Lacks evident sign of toxicity	Nausea (A disorder characterized by a queasy sensation and/or the urge to vomit.)
Primarily subjective with observable component	Has evident sign of toxicity	Diarrhea (A disorder characterized by an increase in frequency and/or loose or watery bowel movements.)
Primarily observable with subjective component	Has evident sign of toxicity	Alopecia (A disorder characterized by a decrease in density of hair compared to normal for a given individual at a given age and body location.)

**Figure 1:**
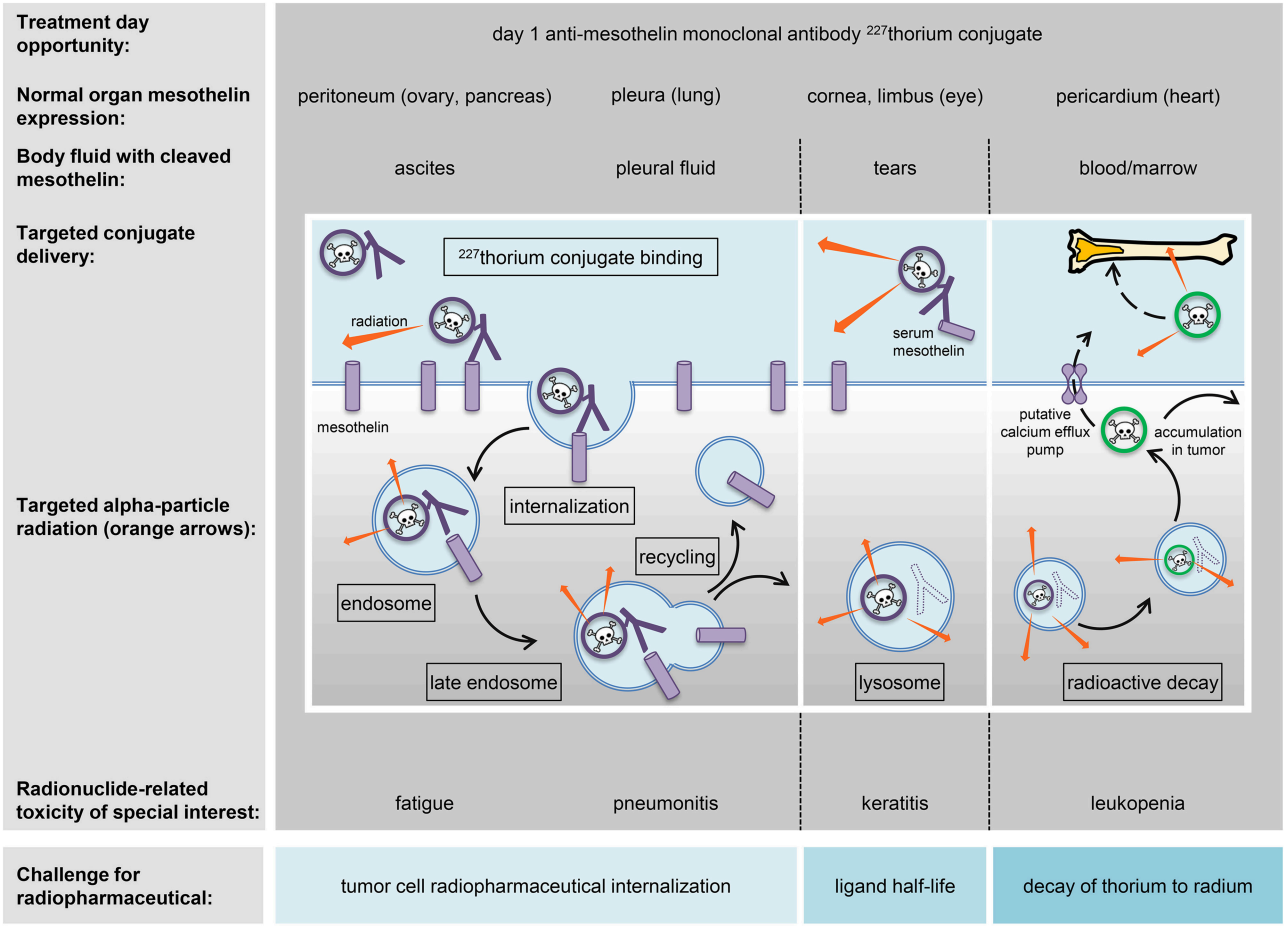
Strategy for radiopharmaceuticals targeting ovarian cancers. A mesothelin-targeted radiopharmaceutical and its anticipated normal organ toxicities are charted in relation to thorium-227 radionuclide delivery and adverse events (toxicities) of special interest. The peritoneum, pleura, cornea/limbus of the eye, and pericardium show molecular expression of mesothelin and are listed together with associated body fluids that might have detectable levels of mesothelin for off-target radiopharmaceutical localization. Marked in boxes are the mechanistic elements of antibody conjugate processing likely to be involved in intended irradiation of tumor cells or in unintended toxicity of normal cells. Challenges for radiopharmaceutical are illustrated and commented upon (blue boxes).

Radiopharmaceuticals might be inhaled, ingested, instilled, or infused by vein. These radioactive drugs aim for accurate and precise molecular delivery of energy-rich radiation to cancer cells either circulating in the blood or in tumors (Figure 1). Radiopharmaceuticals are either neat (i.e., lack a targeting ligand) or conjugated (i.e., have a ligand-linker-payload construct). The radiopharmaceutical radium-223 dichloride falls into the neat class. This is because it is given by vein as a slow bolus solution that tracks to areas of bone turnover as a calcium mimetic without the aid of a ligand (9). In an opposite way, thorium-227-containing radiopharmaceuticals (the parent radionuclide of radium-223) are in the conjugated class. This conjugated molecular entity has three components—a radioactive payload, a linker, and an antibody. Each component possibly contributes to its safety profile. For this reason, NCI has adopted the approach for conjugated radiopharmaceuticals to consider the safety of radionuclide, its cleaved/non-cleaved linker, and “cold” radiopharmaceutical antibody prior to launching clinical trials of the radiopharmaceutical.

Thorium-227 has begun clinical trial testing among women with advanced stage ovarian cancer (NCT03507452). Thorium-227 can be encased by octadentate chelates of the 3,2 hydroxypyridinone (3,2-HOPO) class (13). As an alpha-particle emitting radionuclide, it is a highly potent cytotoxic payload. Given that general knowledge of organ-specific radiation-induced toxicities are sufficient to address safety from radionuclide radioactivity (14), the path for the thorium-227 anti-mesothelin monoclonal antibody conjugate radiopharmaceutical to enter the clinic was relatively straightforward. In a clinical study, a radiopharmaceutical like this might possibly associate with exhaustion, low blood cell or platelet count, gastrointestinal upset, or even specific normal organ toxicity due to the normal expression of the targeted antigen (Figure 1). NCI has reviewed its prior sponsored experience with radiopharmaceuticals in women with advanced stage ovarian cancer (5–8), and considers the toxicity profile of such experimental therapeutic radiopharmaceuticals of acceptable risk for early phase clinical trial study.

The thorium-227 anti-mesothelin monoclonal antibody conjugate radiopharmaceutical uses a 3,2-HOPO chelate to house thorium-227 and link it to a tumor-targeting antibody, and in this case, against mesothelin (13). This antibody has undergone prior clinical development as anetumab ravtansine (15–17). Usual radiochemical purity is 99 percent or greater (13), meaning the 3,2-HOPO chelate linker-antibody is in excess of the thorium-227.

Linker chemistry is important to the distribution of a radioactive payload. While linker as a molecular entity may not contribute to the frequency, severity, or interference of radiopharmaceutical-related toxicity, the stability of the linker does have an impact upon which organs or tissue are affected. Stable non-cleaved linkers restrict payloads to targets for narrow toxicity profiles, whilst less stable cleaved linkers might allow payloads to drift away from targets creating much broader toxicity. Cleavable linkers typically release payloads after processing in endosomes or lysosomes via a variety of mechanisms including acidic degradation, protease cleavage, and thiol-disulfide exchange reactions (Figure 1). The thorium-227 anti-mesothelin monoclonal antibody conjugate radiopharmaceutical is viewed as not having a cleavable linker (13). And so, complete lysosomal degradation of the antibody, alpha-particle mediated damage to the 3,2-HOPO chelate, or loss of decayed daughter radionuclides must manifest for unintended organ or tissue toxicity (Figure 1).

Radiopharmaceutical antibody chemistry demands high affinity target antigen binding for tumor cells and low affinity for normal cells for a realistic therapeutic window. It is therefore desirable for cancer cell-directed antibodies to seek antigens expressed on the surface of those cancer cells prior to internalization and endosomal processing (Figure 1). But, normal cells might also express the target antigen. The antigen mesothelin provides an illustrative example as it is highly expressed in ovarian cancers (16) but also found on the surface of some normal cells like the abdominal peritoneum, lung pleura, eye cornea/limbus, and heart pericardium (17, 18). “Cold” pharmaceutical ligands might have off-target biological effects alone that reflect non-specific or inappropriate target antigen recognition on normal cells. Off-target effects might manifest as toxicity. In the example of thorium-227 anti-mesothelin monoclonal antibody conjugate, the “cold” 3,2-HOPO chelate-antibody pharmaceutical toxicity has not been well-characterized. The ongoing phase I trial in women with advanced stage ovarian cancer should provide this clinical context (NCT03507452).

It bears to reiterate that low level target antigen expression on normal cells may result in specific toxicity, and, cleavage or damage to the linker may induce unintended organ toxicities from free radionuclide. In its effort to study novel radiopharmaceuticals, the NCI has placed an emphasis on a few key pharmacokinetic properties and toxicities (Table 3, Figure 1). One Category C CTCAE example is fatigue. Radiation exposure has been shown to upregulate expression of nucleoside transporters and kinases in cancer cells, perhaps rescuing cells from replication stress (22). It has been speculated that radiation-related exhaustion might result from skeletal muscle expending energy to furnish deoxynucleosides via the bloodstream to irradiated cells demanding their supply for DNA repair. There may be an opportunity to study circulating deoxynucleoside levels further as biomarkers of response in women with chemorefractory advanced stage ovarian cancer treated by radiopharmaceuticals. Anemia, leukopenia or neutropenia, and thrombocytopenia from enhanced elimination or impaired production of marrow constituents, and not circulating mature cells or platelets, are Category A CTCAE acute or cumulative toxicity effects of interest to the NCI. Pneumonitis or eye corneal/limbic keratitis represent two transient, reversible, or persistent Category B CTCAE subacute toxicity effects attributable in a 3-month window following radiopharmaceutical exposure. These are of particular interest to the NCI in its clinical development plans.

**Table 3:** Pharmacokinetic properties of select radiopharmaceuticals.

**Drug product**	**Majority payload**	**Linker**	**Dose** **(MBq)**	**Cmax** **(MBq)**	**Tmax** **(h)**	**T4h** **(%)**	**T24h** **(%)**	**Tp** **(d)**	**Tb** **(d)**	**Te** **(d)**	**Mass dose** (mg)^*^	**Reference**
Radium-223	Alpha particle	NA	3.3	3.3	< 0.25	4	1	11.4	NA	11.4	NA	(19)
Lutetium-177 dotatate	Beta particle	NA	7,400	7,400	< 0.50	7	0	6.7	NA	6.7	NA	(20)
Thorium-227 Anti-Mesothelin MAb^**^	Alpha particle	3,2-HOPO	1.5–7.0	1.5-7.0	< 0.05	NR	13	18.7	4.2	3.3	10–50	(21)

* *Mass dose is the total dose of a non-radioactive or “cold” pharmaceutical, such as the non-cleavable linker and anti-mesothelin antibody of the targeted thorium conjugate radiopharmaceutical. Radium and lutetium dotatate are considered neat radionuclides (i.e., contain no linker-ligand conjugate). Calculations are based on 60-kilogram body weight*.

** *The anti-mesothelin MAb-Thorium-227 conjugate is being tested in a phase 1 first-in-human clinical trial (NCT03507452), and, radiation and antibody mass dose are reported for anticipated dose-escalation range*.

*MBq, megabecquerel; mCi, millicurie; h, hours; d, days; Cmax, maximum serum concentration; Tmax, time after administration when maximum serum concentration is reached; T4H, proportion of administered dose remaining at 4 h after injection; T24H, proportion of administered dose remaining at 24 h after injection; Tp, physical half-life radionuclide; Tb, biological half-life of ligand; Te, effective half calculated as 1/Tp + 1/Tb = 1/Te; mg, milligram; Ref, cited reference; NA, not applicable; NR, not reported; MAb, mesothelin-targeted fully human monoclonal antibody (anetumab, BAY 86-1903); HOPO, octadentate 3, 2-hydroxypyridinone chelator (BAY 1903150)*.

## Perspectives On Radiopharmaceutical Medical Monitoring For Clinical Investigations

The NCI has considered its position on the conduct and the medical monitoring of radiopharmaceutical clinical investigations. The NCI aligns its thoughts on this topic with those guidances provided by the US Food and Drug Administration (23). The NCI considers principal investigator as the responsible leader of a clinical team, meaning they are the individual who both initiates and conducts a clinical investigation and under whose immediate direction an investigational radiopharmaceutical drug is administered or dispensed. No investigator may participate in an NCI-sponsored clinical investigation of a radiopharmaceutical drug until that individual provides the NCI with a completed, signed Statement of Investigator, Form FDA 1572 [21 CFR 312.53(c)]. An investigator personally conducts or supervises a clinical investigation, follows protocol-only changes, ensures that all study staff are informed of protocol-only obligations, informs subjects that radiopharmaceuticals are being used for investigational purposes, ensures informed consent, provides ethics board review, approval and reporting, reports adverse events to NCI as sponsor, reads, and understands the radiopharmaceutical investigator brochure, and maintains adequate and accurate records as well as make those records available for audit by NCI as the sponsor. NCI expects that an investigator administering radiopharmaceuticals complies with all state radiation license regulations and rosters onto a site's radiation authorized user list for the named radiopharmaceutical. NCI also presumes of the radiopharmaceutical investigator study oversight, responsibility for delegation of study tasks or training of study staff, comprehensive supervision inclusive of any third parties. NCI requires a clinical investigator using experimental therapeutics under its sponsored trials to report immediately any adverse event that is alarming (e.g., an unexpected event that is serious or life-threatening) or timely any non-serious adverse events according to its establish NCI timetable recorded in the trial protocol. As trial sponsor, the NCI registers its radiopharmaceutical trials (www.clinicaltrials.gov).

**Figure 2:**
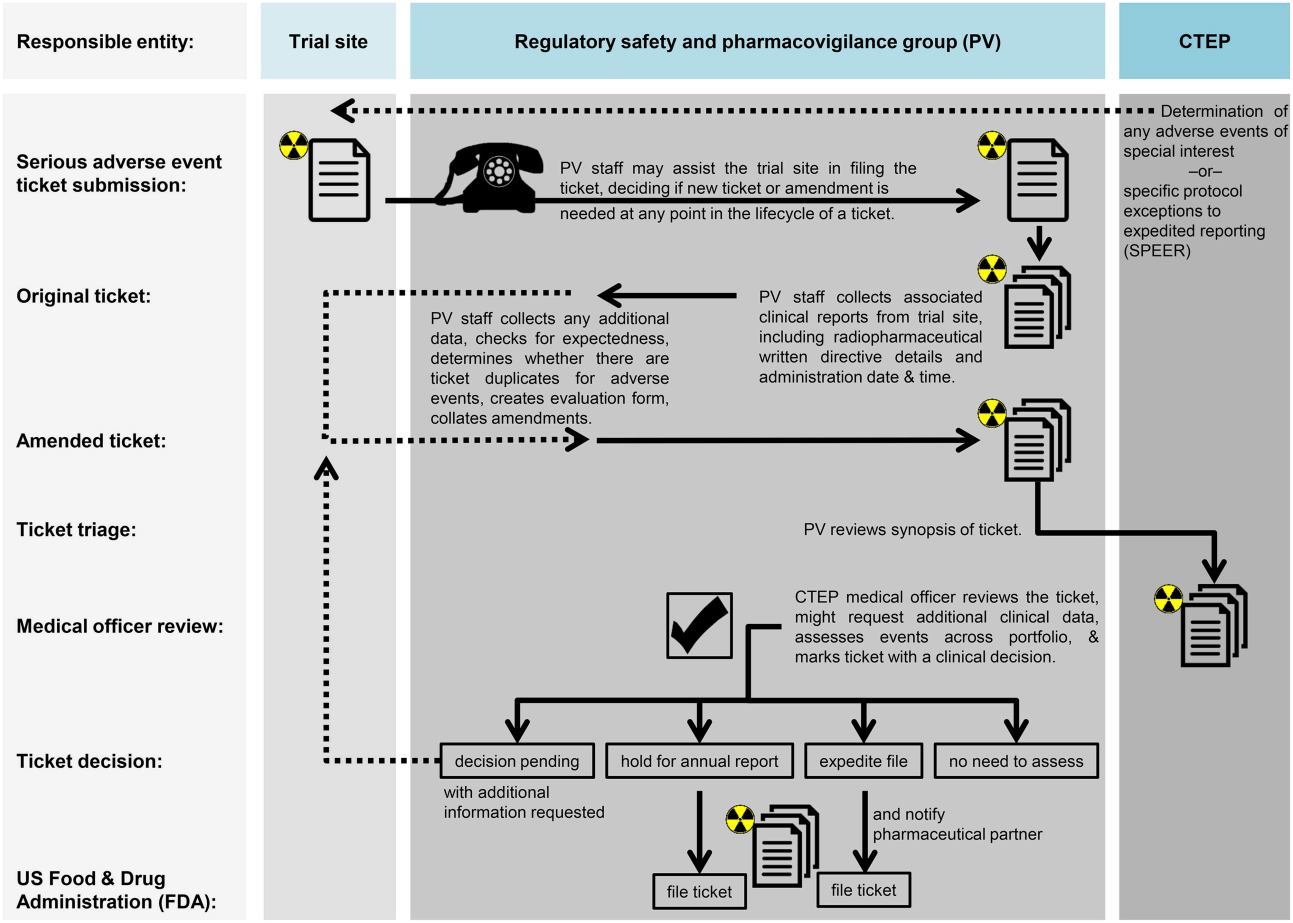
Proposed workflow for monitoring and audit in radiopharmaceutical trials. Proposed workflow steps are charted in relation to the trial site, the regulatory safety and pharmacovigilance group (PV), or the NCI's Cancer Therapy Evaluation Program (CTEP).

The NCI's current thinking for radiopharmaceutical regulatory safety and pharmacovigilance appears in Figure 2. The components of a site's radiopharmaceutical monitoring plan might include—(A) description of each monitoring method employed during the study, how the plan addresses important risks and ensures validity of timing, frequency, logs, and extent of planned monitoring activities as well as definitions of events triggering changes or deviations in planned monitoring activities; (B) communication of monitoring results inclusive of format, content, timing, and archiving requirements for reports and other documentation of monitoring activities from study management and other stakeholders (like site staff, IRB, NCI, FDA), as necessary; (C) processes for addressing unresolved or significant non-compliance with the investigational plan, inclusive of root cause analyses and appropriate corrective and preventive actions for quality management practices applicable to a clinical investigation; (D) description of specific training required for personnel carrying out monitoring activities, including personnel conducting internal data monitoring, statistical monitoring, or other centralized review activities or planned audits of monitoring; and (E) accounting of monitoring plan amendments.

## Conclusion

In summary, this article provides NCI's perspective on radiopharmaceutical clinical development from its vantage of medical monitoring of radionuclide, linker, and ligand toxicity. The current NCI position used prior experience from radiopharmaceutical clinical use in ovarian cancer to sharpen its thinking on conjugated radiopharmaceuticals that might be considered for chemorefractory ovarian cancer patient treatment in the near-term. This article does not outlay NCI's position on inhaled, ingested, or otherwise injected radiopharmaceuticals. Important overarching topics related to regulatory safety and pharmacovigilance such as radiochemical impurity, stability, handling, or distribution are not discussed here. Guidances for some of these topics are found elsewhere (14). Patient and clinical provider education on radiopharmaceuticals remains integral to agent clinical development.

## Ethics Statement

The research presented in this article involved the collection or study of existing data, documents, and records that were publicly available, or the information was recorded by NCI in such a manner that trial subjects cannot be identified directly or through identifiers linked to the subjects. The research is regarded exempt from Institutional Review Board oversight.

## Author Contributions

CK, JC, SF, GS, and SI contributed to the collection and review of any perspective or trial data, analysis, and authentication, and the writing and approval of this manuscript. The views expressed are those of the authors and not those of the U.S. federal government. Links or discussion of specific radiopharmaceutical drug products do not constitute endorsement.

### Conflict of Interest Statement

The authors declare that the research was conducted in the absence of any commercial or financial relationships that could be construed as a potential conflict of interest.
